# Supplementary material for: Association of Metabolic Syndrome with the Risk of Head and Neck Cancer: A 10-Year Follow-Up Study of 10 Million Initially Healthy Individuals
Source: Cancers (Basel). 2023 Aug 15;15(16):4118. doi: 10.3390/cancers15164118 (PMC10452383; doi:10.3390/cancers15164118)
Supplement: Supplementary file 1 [file cancers-15-04118-s001.zip › cancers-2543693-supplementary.pdf]

Table S1. Multivariate Cox proportional hazard model for incidence of head and neck cancer by subtype according to the score of metabolic syndrome components.

Metabolic syndrome Score	No. of patients	Person-years	Incidence rates	Hazard Ratio (95% confidence interval)		
				Model[1]	Model[2]	Model[3]
	**Overall Head Neck Cancer**					
0	1617	21 617 075	0.075	1(reference)	1(reference)	1(reference)
1	2519	21 398 398	0.118	1.57(1.48-1.68)	0.99(0.93-1.06)	0.97(0.91-1.04)
2	2684	16 419 951	0.164	2.18(2.05-2.32)	1.08(1.01-1.15)	1.04(0.98-1.11)
3	2107	11 150 938	0.189	2.52(2.37-2.69)	1.10(1.03-1.17)	1.06(0.99-1.13)
4	1317	6 268 105	0.21	2.81(2.61-3.02)	1.09(1.01-1.18)	1.06(0.98-1.14)
5	488	2 108 426	0.232	3.09(2.79-3.42)	1.13(1.02-1.25)	1.10(0.99-1.22)
	**Laryngeal cancer**					
0	341	21 620 983	0.016	1(reference)	1(reference)	1(reference)
1	659	21 403 979	0.01	1.95(1.71-2.22)	1.06(0.93-1.21)	1.04(0.91-1.19)
2	782	16 425 418	0.048	3.02(2.66-3.43)	1.23(1.08-1.40)	1.20(1.05-1.36)
3	640	11 155 064	0.057	3.63(3.19-4.14)	1.31(1.15-1.50)	1.27(1.11-1.45)
4	413	6 270 633	0.066	4.17(3.61-4.81)	1.34(1.16-1.55)	1.32(1.15-1.53)
5	137	2 109 463	0.065	4.11(3.37-5.01)	1.25(1.03-1.53)	1.26(1.03-1.54)
	**Sino-nasal cancer**					
0	102	21 621 806	0.005	1(reference)	1(reference)	1(reference)
1	108	21 405 885	0.005	1.07(0.82-1.40)	0.73(0.56-0.96)	0.72(0.55-0.94)
2	135	16 427 587	0.008	1.74(1.35-2.25)	0.96(0.74-1.25)	0.93(0.71-1.21)
3	96	11 156 990	0.09	1.82(1.38-2.41)	0.88(0.66-1.17)	0.85(0.63-1.13)
4	63	6 271 885	0.01	2.12(1.55-2.91)	0.91(0.66-1.26)	0.88(0.64-1.22)
5	35	2 109 853	0.017	3.51(2.39-5.15)	1.39(0.94-2.06)	1.35(0.91-2.00)
	**Hypopharyngeal cancer**					
0	118	21 621 807	0.006	1(reference)	1(reference)	1(reference)
1	219	21 405 769	0.01	1.87(1.50-2.34)	0.98(0.78-1.23)	0.95(0.76-1.19)
2	244	16 427 397	0.015	2.71(2.18-3.38)	1.06(0.85-1.32)	1.01(0.81-1.26)
3	199	11 156 747	0.018	3.25(2.59-4.09)	1.11(0.88-1.40)	1.06(0.84-1.33)
4	114	6 271 792	0.018	3.31(2.56-4.29)	1.00(0.77-1.29)	0.97(0.75-1.26)
5	35	2 109 859	0.017	3.02(2.07-4.40)	0.85(0.58-1.24)	0.85(0.58-1.24)
	**Oropharyngeal cancer**					
0	241	21 621 375	0.011	1(reference)	1(reference)	1(reference)
1	466	21 404 722	0.022	1.95(1.67-2.28)	1.24(1.06-1.45)	1.22(1.04-1.43)
2	492	16 426 570	0.03	2.69(2.30-3.14)	1.34(1.15-1.57)	1.31(1.12-1.53)
3	334	11 156 199	0.03	2.69(2.28-3.17)	1.20(1.01-1.42)	1.17(0.99-1.38)
4	204	6 271 476	0.032	2.92(2.42-3.52)	1.19(0.98-1.44)	1.16(0.96-1.40)
5	77	2 109 700	0.037	3.28(2.53-4.23)	1.27(0.98-1.65)	1.25(0.96-1.62)
	**Oral cancer**					

0	357	21 621 025	0.017	1(reference)	1(reference)	1(reference)
1	509	21 404 672	0.024	1.44(1.26-1.65)	1.01(0.88-1.15)	0.99(0.87-1.14)
2	534	16 426 495	0.033	1.97(1.72-2.25)	1.11(0.97-1.27)	1.09(0.95-1.25)
3	440	11 156 064	0.039	2.39(2.08-2.75)	1.18(1.02-1.36)	1.15(0.99-1.33)
4	283	6 271 301	0.045	2.74(2.34-3.20)	1.20(1.02-1.40)	1.17(0.99-1.38)
5	102	2 109 689	0.048	2.93(2.35-3.65)	1.17(0.94-1.47)	1.15(0.92-1.44)
	**Nasopharyngeal cancer**					
0	226	21 621 331	0.011	1(reference)	1(reference)	1(reference)
1	262	21 405 277	0.012	1.17(0.98-1.40)	0.86(0.72-1.03)	0.86(0.71-1.03)
2	256	16 427 076	0.016	1.49(1.24-1.78)	0.92(0.77-1.12)	0.91(0.76-1.10)
3	197	11 156 632	0.018	1.68(1.39-2.04)	0.96(0.79-1.17)	0.95(0.78-1.16)
4	114	6 271 726	0.018	1.73(1.38-2.17)	0.93(0.74-1.17)	0.92(0.73-1.17)
5	46	2 109 782	0.022	2.08(1.51-2.85)	1.08(0.78-1.50)	1.07(0.78-1.49)
	**Salivary gland cancer**					
0	247	21 621 273	0.011	1(reference)	1(reference)	1(reference)
1	320	21 405 105	0.015	1.31(1.11-1.55)	1.02(0.86-1.21)	1.01(0.85-1.20)
2	272	16 427 046	0.017	1.45(1.22-1.72)	0.96(0.80-1.15)	0.95(0.79-1.13)
3	230	11 156 463	0.021	1.81(1.51-2.16)	1.07(0.88-1.29)	1.05(0.87-1.27)
4	144	6 271 580	0.023	2.01(1.64-2.47)	1.07(0.86-1.33)	1.05(0.85-1.31)
5	60	2 109 752	0.028	2.49(1.88-3.31)	1.22(0.91-1.64)	1.20(0.90-1.61)

Incidence rate per 1000 person-years. Model [1]: Unadjusted. Model [2]: Adjusted for age, gender, smoking, alcohol consumption, regular exercise and income. Model [3]: Adjusted for age, gender, smoking, alcohol consumption, regular exercise, income, diabetes and hypertension.

Table S2. Multivariate Cox proportional hazard model for incidence of head and neck cancer by subtype according to the presence or absence of the metabolic syndrome components.

	Laryngeal cancer				Sino-nasal cancer				Hypopharyngeal cancer				Oropharyngeal cancer			
Parameter	EVENT	Person - years	Incidence rates	HR (95% CI)	EVENT	Person - years	Incidence rates	HR (95% CI)	EVENT	Person - years	Incidence rates	HR (95% CI)	EVENT	Person - years	Incidence rates	HR (95% CI)
High waist circumference																
Yes	772	15 442 965	0.05	1.05(0.96-1.14)	383	15 444 097	0.01	1.23(1.02-1.48)	760	15 444 119	0.011	0.64(0.55-0.76)	1352	15 443 108	0.03	1.04(0.94-1.16)
No	2200	63 546 283	0.029	1 (reference)	156	63 549 911	0.006	1 (reference)	169	63 549 254	0.012	1 (reference)	462	63 546 936	0.021	1 (reference)
High fasting glucose																
Yes	1420	24 548 785	0.058	1.13(1.05-1.22)	308	24 550 425	0.009	1.12(0.95-1.34)	492	24 550 103	0.018	1.07(0.94-1.22)	1018	24 548 674	0.032	1.09(0.99-1.20)
No	1552	54 438 752	0.029	1 (reference)	231	54 443 583	0.006	1 (reference)	437	54 443 270	0.009	1 (reference)	796	54 441 370	0.019	1 (reference)
High blood pressure																
Yes	2005	34 149 956	0.059	1.21(1.12-1.31)	213	34 156 283	0.01	1.15(0.95-1.38)	278	34 155 649	0.019	1.27(1.10-1.47)	730	34 154 005	0.032	1.04(0.94-1.15)
No	967	44 834 795	0.022	1 (reference)	326	44 837 725	0.005	1 (reference)	651	44 837 723	0.006	1 (reference)	1084	44 836 039	0.016	1 (reference)
High triglyceride																
Yes	1412	27 600 382	0.051	1.13(1.05-1.21)	330	27 604 964	0.008	0.85(0.72-1.01)	507	27 604 505	0.015	1.04(0.92-1.19)	1004	27 603 091	0.029	1.04(0.95-1.15)
No	1560	51 384 369	0.03	1 (reference)	209	51 389 044	0.006	1 (reference)	422	51 388 868	0.01	1 (reference)	810	51 386 954	0.02	1 (reference)
Low HDL cholesterol																
Yes	871	21 610 338	0.04	1.08(0.99-1.17)	368	21 613 073	0.008	1.01(0.84-1.22)	673	21 612 895	0.012	0.98(0.85-1.14)	1313	21 611 990	0.023	0.95(0.86-1.06)
No	2101	57 374 414	0.036	1 (reference)	171	57 380 935	0.006	1 (reference)	256	57 380 478	0.012	1 (reference)	501	57 378 054	0.023	1 (reference)

HRs, hazard ratios; HDL, high density lipoprotein. Incidence rates per 1000 person-years. Adjusted for age, gender, smoking, alcohol consumption, regular exercise, income, diabetes and hypertension.

Parameter	Oral cancer				Nasopharyngeal cancer				Salivary gland cancer			
	Event	Person - years	Incidence rates	HR (95% CI)	Event	Person -years	Incidence rates	HR (95% CI)	Event	Person -years	Incidence rates	HR (95% CI)
High waist circumference												
Yes	563	15 442 691	0.036	1.02(0.93-1.13)	811	15 443 575	0.019	1.18(1.03-1.35)	934	15 443 386	0.022	1.17(1.03-1.33)
No	1662	63 545 579	0.026	1 (reference)	290	63 548 250	0.013	1 (reference)	339	63 547 835	0.015	1 (reference)
High fasting glucose												
Yes	874	24 548 320	0.036	0.99(0.90-1.08)	689	24 549 702	0.017	0.96(0.85-1.09)	796	24 549 518	0.019	1.01(0.90-1.13)
No	1351	54 439 949	0.025	1 (reference)	412	54 442 123	0.013	1 (reference)	477	54 441 704	0.015	1 (reference)
High blood pressure												
Yes	1322	34 153 003	0.039	1.12(1.02-1.22)	520	34 155 288	0.017	0.96(0.85-1.09)	590	34 154 902	0.02	1.01(0.90-1.14)
No	903	44 835 267	0.02	1 (reference)	581	44 836 537	0.012	1 (reference)	683	44 836 319	0.013	1 (reference)
High triglyceride												
Yes	1002	27 602 315	0.036	1.12(1.03-1.22)	644	27 604 068	0.017	0.99(0.88-1.12)	772	27 603 820	0.018	0.96(0.85-1.07)
No	1223	51 385 955	0.024	1 (reference)	457	51 387 757	0.013	1 (reference)	501	51 387 401	0.015	1 (reference)
Low HDL cholesterol												
Yes	778	21 611 117	0.036	1.14(1.04-1.24)	790	21 612 508	0.014	1.00(0.87-1.14)	843	21 612 045	0.02	1.08(0.96-1.22)
No	1447	57 377 152	0.025	1 (reference)	311	57 379 317	0.014	1 (reference)	430	57 379 176	0.015	1 (reference)
